# Propylthiouracil-Induced Neonatal Goiter: An Avoidable Problem

**DOI:** 10.7759/cureus.40389

**Published:** 2023-06-13

**Authors:** Keyur Saboo, Rinkle R Gemnani, Nishtha Manuja, Sunil Kumar, Sourya Acharya

**Affiliations:** 1 Department of Medicine, Jawaharlal Nehru Medical College, Wardha, IND; 2 Department of Internal Medicine, Jawaharlal Nehru Medical College, Wardha, IND

**Keywords:** pregnancy, mithimazole, neonatal goiter, maternal hyperthyroidism, propylthiouracil

## Abstract

We present a case of neonatal goiter caused by exposure to propylthiouracil (PTU) during pregnancy in this case report. The mother was treated with PTU while pregnant and had a history of Graves' illness. The baby had a neck tumor and was having breathing problems when they were first seen. A big thyroid gland was detected by a neck CT scan. This instance serves as a reminder of the significance of managing maternal thyroid problems appropriately during pregnancy as well as the requirement for careful thyroid function monitoring in newborns exposed to PTU. Given the possible hazards to the growing baby, it also calls into question whether PTU is an effective first-line treatment for maternal Graves' illness during pregnancy. This case report highlights the importance of shared decision-making with patients (by discussing different treatment options and their side effects).

## Introduction

Neonatal goitrous hypothyroidism has been linked to the treatment of Graves' illness in pregnant women with propylthiouracil (PTU). Moreover, thyrotropin (TSH) receptor-blocking antibodies can result in embryonic hypothyroidism without goiter, while thyroid-stimulating immunoglobulin (TSIG) can cross through the placenta and produce fetal hyperthyroidism. It is challenging to predict how these substances may affect prenatal thyroid function; they may cause everything from hypothyroidism to hyperthyroidism in terms of neonatal thyroid dysfunction. Severe cases of prenatal thyroid enlargement should be considered when assessing the differential diagnosis of a neonatal neck mass since they can affect respiratory function [1]. Pregnancy-related hyperthyroidism should be treated with caution due to the possibility of adverse effects on both the mother and the unborn child. PTU is a medication that is used to treat hyperthyroidism in pregnant women. PTU's most frequent side effects include nausea and vomiting, loss of taste or appetite, joint discomfort, and dizziness. As they are often little, patients frequently go away on their own without any advice from the treating physician. More severe adverse effects are agranulocytosis, a dangerous disorder marked by a sharp decline in white blood cell count. PTU was linked to an elevated incidence of neonatal congenital malformations including situs inversus dextrocardia, an isolated unilateral kidney, and cardiac outflow tract problems, according to recent investigations. Pregnant women using PTU need to be regularly watched by their healthcare provider due to these possible side effects. In general, the benefits of using PTU to treat hyperthyroidism during pregnancy outweigh the risks of side effects, but it is important to weigh these risks carefully with the help of a healthcare provider. PTU should be switched to methimazole during the second trimester of pregnancy [2] as methimazole is considered safer during the second trimester of pregnancy.

## Case presentation

**Figure 1:**
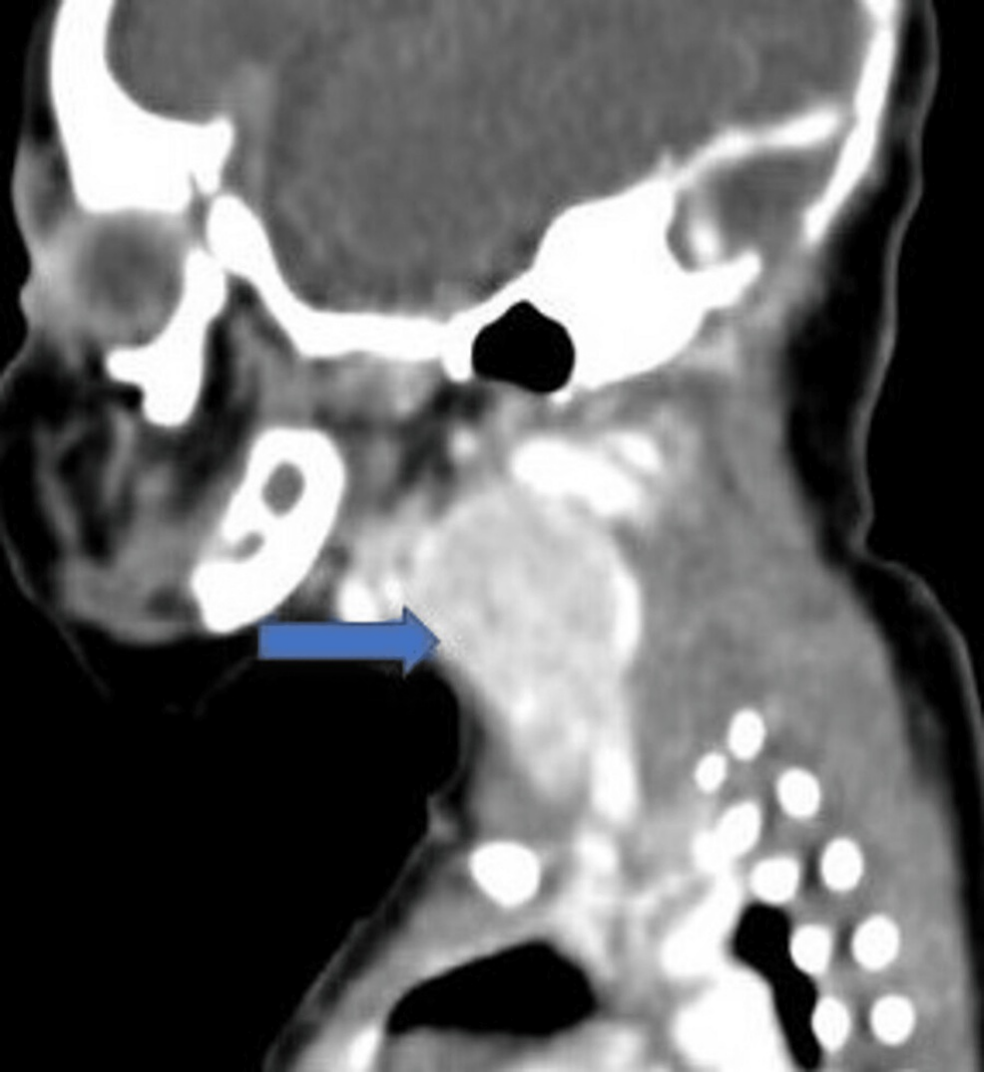
CECT neck showing the enlarged thyroid gland in the neonate (blue arrow)

**Figure 2:**
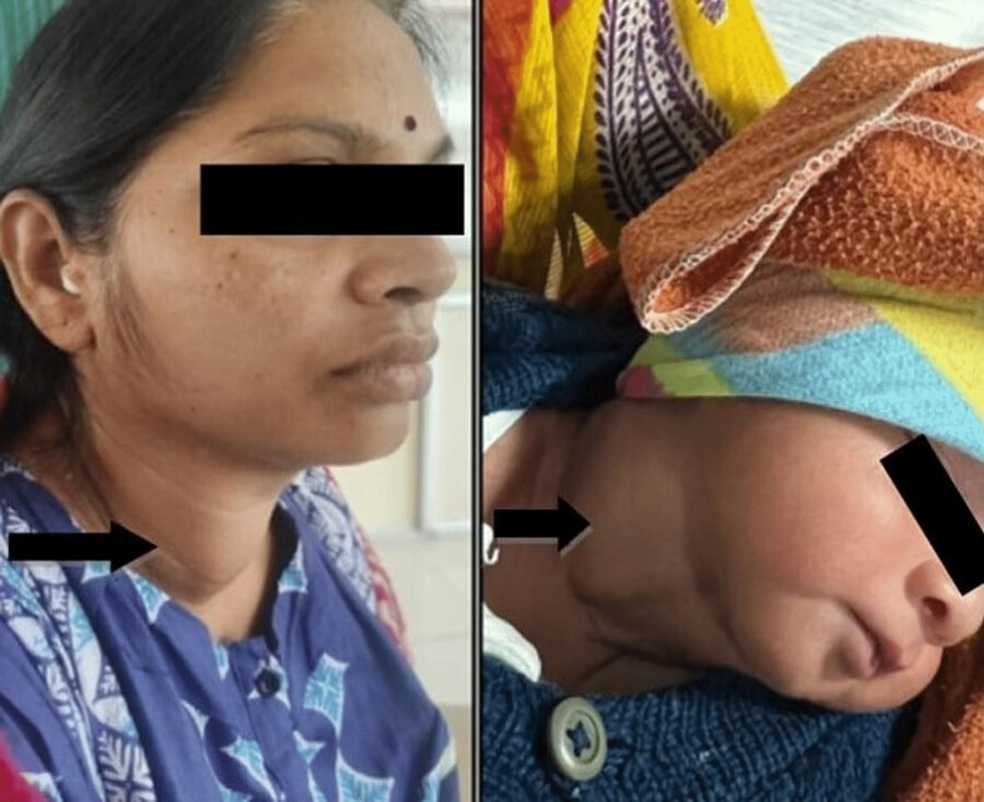
Image showing goiter in mother as well as neonatal goiter (black arrow)

We present a case of neonatal goiter caused by PTU exposure during pregnancy in this case report. The mother was treated with PTU (300 mg/day in three divided doses) while pregnant and had a history of Graves' illness. The baby had a neck tumor and was experiencing breathing problems when first seen. Ultrasonography of the neck was done which was suggestive of a thyroid nodule; hence, to confirm, a CT neck was advised which revealed a large thyroid gland (Figure 1). This case serves as a reminder of the significance of managing maternal thyroid problems appropriately during pregnancy and the need for careful thyroid function monitoring in newborns exposed to PTU (Table 1). Given the potential hazards to the developing baby, it also raises questions about whether PTU is an effective first-line treatment for maternal Graves' illness during pregnancy (Figure 2). This case report also highlights the importance of considering alternative treatment options such as methimazole for maternal Graves' illness during pregnancy. Methimazole is another antithyroid drug that is considered safer during the second and third trimesters. However, sometimes methimazole can also cross the placenta and can cause hypothyroidism in the newborn.

**Table 1:** Investigation profile of the patient

Investigations	Mother	Neonate	Reference Values
Hemoglobin	11.2 g/dl	17 g/dl	13-17 g/dl
Serum Creatinine	0.6 mg/dl	0.5 mg/dl	0.5-1.2 mg/dl
Albumin	3.2 g/dl	-	3.5-5.0 g/dl
Aspartate Aminotransferase	25 U/L	-	<50 U/L
Alanine Aminotransferase	30 U/L	-	17-59 U/L
Total Bilirubin	0.5 mg/dl	-	0.2-1.3 mg/dl
Free Triiodothyronine 3 [Ft3]	2.84 ng/ml	2.75 ng/ml	2.77-5.27 ng/ml
Free Triiodothyronine 4 [Ft4]	0.81 ng/ml	1.1 ng/ml	0.78-2.19 ng/ml
Thyroid-Stimulating Hormone [Tsh]	4.26 µIU/ml	2.18 µ IU/ml	0.465-4.68µ IU/ml
Anti Tpo Antibody	46 IU/ml	-	< 50 IU/ml

## Discussion

Pregnancy with hyperthyroid disease should continue their antithyroid therapy but should be closely monitored by a medical expert. To ensure effective monitoring of the child after birth, the treating physician should be aware of the prenatal maternal diagnosis and treatment plan. T4 and TSH levels should be assessed on the second and seventh days of life, respectively, with further testing based on the neonatal clinical condition and the findings of earlier tests. Imaging of the neck like ultrasonography, MRI, or CT scans should be advised to determine the thyroid size in infants who were exposed to anti-thyroid drugs during pregnancy and who have neck masses and respiratory symptoms [3,4]. In both the prenatal and postnatal phases, ultrasonography is thought to be the preferred imaging technique for assessing fetal thyroid. In situations of prenatal thyroid problems, careful observation and adequate medical intervention are crucial to achieving the best possible outcomes for both mother and child.

While treatment with PTU is generally considered safe, it can have potentially negative effects on the developing fetus if not managed appropriately. PTU should be used in the first trimester of pregnancy then switched over to methimazole. Neonatal goiter had been reported by Li et al. and Clementi et al. as a result of the PTU treatment, and the probable hypothesis they proposed was due to crossing the placenta and suppressing the fetal thyroid gland [5,6]. The net effect of the maternal to fetal transfer of PTU, stimulating antibodies, and blocking antibodies are difficult to explain. Neonatal thyroid dysfunction can range from frank hypothyroidism to hyperthyroid neonatal goiter secondary to exposure to maternal thyroid-stimulating immunoglobulin [6]. It is also worth noting that there are alternative treatments for hyperthyroidism that may be safer for use during pregnancy. For example, methimazole is another medication commonly used for hyperthyroidism, and studies have suggested that it may have a lower risk of crossing the placenta and affecting the fetal thyroid gland. However, evidence from these studies is lacking to choose between PTU followed by methimazole and vice versa. Nowadays, antithyroid prescription patterns are not necessary during pregnancy if follow-up is prompt [7-8].

Congenital goiter has been reported in areas of endemic goiter and sporadically elsewhere. The probable hypothesis may be due to athyreosis because of the defect in the embryologic development of the thyroid gland or the destruction of the fetal thyroid by radioiodine given to the mother during the last trimester. Sometimes, this may occur due to the destruction of the thyroid gland by autoimmune diseases such as Hashimoto's thyroiditis [9]. Sporadic neonatal goiter may also be due to inborn errors of thyroid hormonogenesis, peripheral tissue resistance to the action of thyroid hormones, and gestational consequences of maternal ingestion of goitrogens like PTU and methimazole which crosses the placenta. Treatment of thyrotoxic mothers with antithyroid drugs only infrequently results in neonatal goiter. There are reports by Li et al. regarding neonatal goiter in mothers who were treated with iodide though less common. They have also reported antithyroid drugs causing thyroid hyperplasia in human fetuses by stimulation of fetal pituitary thyrotropin [10].

This case report highlights the recommendation of the lowest dose of an antithyroid drug maintaining the maternal serum levels of free thyroid hormones in the upper limits of normal or even in the low thyrotoxic range in thyrotoxic pregnant women. By this, infants will be exposed to the least amount of antithyroid drugs. An evaluation of the newborn infant by measurement of serum-free T4 and TSH in cord blood or in blood obtained during the early days of neonatal life is valuable.

## Conclusions

The case study of newborn goiter caused by PTU emphasizes the significance of treating maternal thyroid problems correctly throughout pregnancy. To minimize preventable consequences like newborn goiter, healthcare professionals must carefully weigh the possible risks and advantages of pharmaceutical alternatives for pregnant women with thyroid issues. Patient follow-ups can also play an important role in better outcomes. Monitoring the fetal thyroid function with regular ultrasounds and conducting cord blood thyroid hormone levels may be useful in preventing or identifying this type of complication.
